# Durable clinical and immunologic response to an off-the-shelf EWSR1–FLI1 peptide vaccine in metastatic Ewing sarcoma

**DOI:** 10.1038/s41698-026-01642-4

**Published:** 2026-08-08

**Authors:** Branko Calukovic, Katrin Benzler, Henning Zelba, Christian Seitz, Simone Kayser, Magdalena Feldhahn, Borong Shao, Martin Schulze, Olga Maksimovic, Veit Scheble, Florian Battke, Lars Zender, Ulrich M. Lauer, Saskia Biskup, Christoph K. W. Deinzer

**Affiliations:** 1 Department of Internal Medicine VIII - Medical Oncology and Pneumology, University Hospital Tübingen, Tübingen, Germany; 2 Center for Soft Tissue Sarcomas, GIST and Bone Tumors (ZWS) of the University Hospital Tübingen and the Comprehensive Cancer Center (CCC) Tübingen-Stuttgart, Tübingen, Germany; 3 DFG Cluster of Excellence 2180 ‘Image-Guided and Functional Instructed Tumor Therapy’ (iFIT), University of Tübingen, Tübingen, Germany; 4 Center for Human Genetics Tübingen, Tübingen, Germany; 5 Hopp Children’s Cancer Center Heidelberg (KiTZ), Heidelberg University Hospital and German Cancer Research Center (DKFZ), Heidelberg, Germany; 6 Department of Pediatric Oncology, Hematology, and Immunology, Heidelberg University Hospital, Heidelberg, Germany; 7 Department of General Pediatrics, Hematology and Oncology, University Children’s Hospital, Tübingen, Germany; 8 CeGaT GmbH, Tübingen, Germany; 9 MVZ Oncology Tübingen, Tübingen, Germany; 10 German Cancer Consortium (DKTK), German Cancer Research Center (DKFZ), Tübingen, Germany

**Keywords:** Cancer, Immunology, Oncology

## Abstract

Ewing sarcoma is a rare, fusion-driven malignancy with poor prognosis in the metastatic setting, for which no established immunotherapeutic treatment is currently available. Fusion breakpoints are rational precision immunotherapy targets, yet clinical evidence of immunogenicity is scarce. We administered an off-the-shelf multi-peptide vaccine spanning the type 1 EWSR1–FLI1 breakpoint to a patient with high-burden metastatic Ewing sarcoma following multimodal therapy. Vaccinations were combined with GM-CSF and topical imiquimod. Longitudinal immune monitoring by in vitro peptide stimulation and intracellular cytokine staining revealed de novo polyfunctional CD4⁺ T-cell responses against all four fusion-derived peptides, first detectable by month 7 and persisting beyond two years. Treatment was well tolerated with only grade 1 local reactions. Durable disease stability was maintained for more than 26 months. These first-in-human data support the feasibility, safety, and immunogenicity of a fusion-derived peptide vaccine and warrant further evaluation of precision immunotherapy in sarcomas driven by recurrent gene fusions.

## Introduction

Ewing sarcoma is a rare and aggressive malignancy, with an incidence of about one case per million and a peak between 10 and 20 years of age^1^. It most often arises in the bones but may also occur in soft tissues. The disease is driven by recurrent chromosomal translocations, most frequently the EWSR1–FLI1 fusion, resulting in an aberrant transcription factor that drives oncogenesis.

Current standard treatment is multimodal, combining multi-agent chemotherapy, surgery and/or radiotherapy for local control, and consolidation. In Europe, the VIDE regimen (vincristine, ifosfamide, doxorubicin, etoposide) was most commonly applied, while in North America alternating vincristine, doxorubicin, and cyclophosphamide with ifosfamide and etoposide (VDC/IE) was preferred. In the meantime, the randomized EE2012 trial demonstrated that VDC/IE achieves superior event-free survival with less toxicity and shorter treatment duration compared to VIDE, establishing the VDC/IE regimen as the standard of care in both North America and Europe^2^. Radiotherapy also plays an essential role, given the intrinsic radiosensitivity of Ewing sarcoma, and stereotactic body radiotherapy (SBRT) is increasingly used for metastatic lesions^3^. Despite these intensive regimens, the prognosis for patients with metastatic or relapsed disease remains poor, with five-year survival rates under 30%^4^.

Prognosis is influenced not only by tumor burden and response to chemotherapy but also by host immunity. The degree of histologic necrosis following induction therapy is an established predictor of outcome^5^. Moreover, early lymphocyte recovery after chemotherapy has been associated with improved survival in Ewing sarcoma and other pediatric sarcomas^6–9^. These findings support the notion that effective antitumor immunity contributes to disease control.

In this context, immunotherapy represents an attractive but as yet unrealized strategy. Ewing sarcoma typically exhibits low mutational burden and a relatively “cold” tumor microenvironment, limiting efficacy of immune checkpoint inhibitors. Peptide-based vaccines are designed to stimulate tumor-specific T-cell responses. They may be personalized, e.g., RNA neoantigen vaccines^10^, or standardized against shared tumor-associated antigens^11^. The recurrent and tumor-specific EWSR1–FLI1 breakpoint creates a unique, fusion-derived neoepitope absent from normal tissues, providing a compelling precision oncology target.

Previous vaccination attempts in Ewing sarcoma employed complex, individualized strategies, such as dendritic cell pulsing or genetically modified autologous tumor cells secreting GM-CSF^12,13^. These approaches proved feasible but were limited by logistical challenges. Advances in peptide manufacturing and immune monitoring now allow simpler, off-the-shelf multi-peptide vaccines targeting recurrent fusions. Encouraging results have been reported in glioblastoma and IDH1-mutant glioma, where peptide vaccines induced durable, polyfunctional T-cell responses^14–17^. Conceptual reviews highlight such vaccines as a strategy to “build a bridge over troubled waters” in difficult-to-treat cancers^18^.

Here, we report the case of an adult patient with high-burden metastatic Ewing sarcoma who received an off-the-shelf multi-peptide vaccine spanning the canonical EWSR1–FLI1 fusion breakpoint following multimodal therapy. We demonstrate that this strategy was safe, induced robust and durable polyfunctional CD4⁺ T-cell responses, and coincided with long-term clinical stability exceeding two years. This first-in-human experience highlights the feasibility and translational potential of fusion-derived peptide vaccines as a novel precision immunotherapy strategy for patients with Ewing sarcoma.

## Results

### Case description

A 30-year-old male with no comorbidities presented in January 2022 with progressive pain of the left upper arm and shoulder. MRI revealed a large proximal humeral lesion with intramedullary extension, cortical breakthrough, and an extensive soft tissue component, consistent with a high-burden primary tumor. The primary tumor was located in the proximal left humerus and measured 11 × 3.8 × 4.4 cm on baseline imaging. Initial staging by CT and 18F-FDG PET demonstrated multifocal osseous metastatic disease without pulmonary metastases, with documented skeletal involvement of the left humerus, C2, C4, T1, T10, T11, and the ilium. Skip metastases were also documented in the distal left humerus. The initial clinical stage was cT3 Nx M1b, corresponding to stage IVB according to UICC/AJCC. Histopathology demonstrated a small round blue cell tumor, and molecular profiling confirmed the presence of a type 1 EWSR1–FLI1 fusion, consistent with Ewing sarcoma^1^. Immunohistochemistry showed positivity for FLI1 and CD99, while EMA, desmin, S100, cytokeratin, smooth muscle actin, synaptophysin, and SSX were negative. Additional sequencing identified two stop-gain mutations in KMT2D, the clinical relevance of which remains uncertain. Despite the high radiographic tumor burden, serum lactate dehydrogenase (LDH) was not elevated at baseline, measuring 171 U/L on 26 January 2022, below the institutional upper limit of normal of 250 U/L. During longitudinal monitoring up to the February 2025 manuscript data cut-off, LDH remained within the institutional reference range, with a maximum value of 246 U/L recorded on 9 March 2022. After completion of chemotherapy and radiotherapy and shortly before vaccination initiation, LDH was 149 U/L on 23 November 2022. The patient underwent intensive multimodal therapy according to contemporary Ewing sarcoma treatment standards. Induction chemotherapy consisted of six cycles of VIDE between January and May 2022, comprising vincristine on day 1 and doxorubicin, etoposide phosphate, and ifosfamide on days 1–3. Cycles 1–4 were administered at full protocol dose for doxorubicin, etoposide phosphate, and ifosfamide: doxorubicin 20 mg/m²/day, etoposide phosphate 150 mg/m²/day, and ifosfamide 3000 mg/m²/day on days 1–3. Vincristine was administered at 2 mg on day 1 of each cycle, corresponding to the institutional absolute dose cap. After febrile neutropenia with pronounced mucositis and pancytopenia following VIDE cycle 4, doxorubicin, etoposide phosphate, and ifosfamide were reduced to 80% of the protocol dose from cycle 5 onward, corresponding to doxorubicin 16 mg/m²/day, etoposide phosphate 120 mg/m²/day, and ifosfamide 2400 mg/m²/day on days 1–3. The approximate delivered cumulative VIDE doses were vincristine 12 mg absolute dose, doxorubicin 336 mg/m², etoposide phosphate 2520 mg/m², and ifosfamide 50.4 g/m². Restaging after completion of induction chemotherapy demonstrated stable disease with local regression of the primary tumor. The patient subsequently received six cycles of VAI consolidation chemotherapy between June and October 2022, consisting of vincristine, dactinomycin, and ifosfamide. VAI cycle 1 was administered at full protocol dose for dactinomycin and ifosfamide, with dactinomycin 0.75 mg/m²/day and ifosfamide 3000 mg/m²/day on days 1–2. From VAI cycle 2 onward, dactinomycin and ifosfamide were reduced to 80% of the protocol dose because of prior febrile neutropenia and ongoing hematologic toxicity, corresponding to dactinomycin 0.6 mg/m²/day and ifosfamide 2400 mg/m²/day when administered. Vincristine was given at 2 mg in VAI cycles 1–4 and reduced to 1 mg in cycles 5–6. After three cycles of VAI, intra-articular resection of the proximal left humerus was performed on 18 August 2022, including resection of a 16.5 cm tumor-bearing bone segment and reconstruction with an inverse proximal humeral MUTARS endoprosthesis. The postoperative course was uncomplicated, with regular implant positioning on postoperative radiography. A formal histopathologic regression score, quantified tumor necrosis percentage, and resection-margin status were not available from the reviewed documentation. Three additional VAI cycles were then administered. During the final VAI cycle, administered from 17 to 18 October 2022, dactinomycin was omitted because of concurrent radiotherapy. Consolidative radiotherapy was delivered from 6 October to 17 November 2022 as part of multimodal local and metastatic-site control^19^. Intensity-modulated radiotherapy was applied to the primary tumor region of the left humerus with 50.4 Gy in 28 fractions of 1.8 Gy using 6 MV photons. Additional osseous metastatic sites were treated with 45 Gy in 1.8 Gy fractions, including lesions in the cervical and upper thoracic spine (C1–T2), thoracolumbar/pelvic target regions including T10–T11 and L1–L5, and the sternum. The sternal lesion was treated with photons up to 19.8 Gy followed by continuation with 8 MeV electrons to a cumulative dose of 45 Gy. Radiotherapy to the osseous metastatic sites was temporarily paused on selected treatment days because of pronounced pancytopenia and lymphocytopenia requiring transfusional support and G-CSF, while irradiation of the primary tumor region was not interrupted. Radiotherapy was completed on 17 November 2022. Acute radiotherapy-associated toxicity included grade 2 epitheliolysis in the left axillary region, mild erythema over the sternum, mild dysphagia, oral dryness, dysgeusia, appetite loss, mild weight loss, and transient diarrhea with intermittent blood admixture. The latter led to short inpatient evaluation after completion of radiotherapy. Colonoscopy showed nonspecific colitis without active bleeding. This episode occurred before initiation of peptide vaccination. Despite intensive multimodal therapy, prognosis at this stage remained poor, as long-term progression-free survival beyond two years is rarely achieved in patients with multifocal metastatic Ewing sarcoma^20^.

Peptide vaccination was initiated on 2 December 2022, 15 days after completion of radiotherapy. The priming phase consisted of four vaccinations within seven days, administered on 2, 5, 7, and 8 December 2022, followed by regular booster vaccinations. Most vaccinations were co-administered with subcutaneous sargramostim (GM-CSF, 83 µg) and topical imiquimod, as described in the “Methods” section and Supplementary Information. From October 2023 onward, a second vaccine batch was used. At the February 2025 manuscript data cut-off, the patient had received 23 vaccinations over 26 months, consisting of the initial priming phase followed by 19 booster vaccinations. Treatment was well tolerated, with only grade 1 local reactions (erythema, swelling, induration) reported. No systemic vaccine-associated adverse events or clinically relevant immune-mediated toxicities were observed.

Serial imaging demonstrated durable disease control during follow-up. Staging in January 2023 showed stable disease without new metastatic lesions. In March 2023, whole-body MRI no longer showed clearly recognizable osseous metastases, and CT of the thorax did not reveal new suspicious lesions. Subsequent imaging in July and October 2023 remained without evidence of new malignancy-suspicious disease. In January 2024, MRI of the left upper arm and whole-body MRI again showed no evidence of local recurrence or newly detectable metastatic lesions, and previously documented osseous metastases were no longer clearly demarcated morphologically. At clinical assessment in January 2024, the patient remained clinically stable with ECOG performance status 0 and Karnofsky performance status 90%. At the February 2025 manuscript data cut-off, more than three years after initial diagnosis and 26 months after initiation of peptide vaccination, the patient remained clinically well with ECOG performance status 0 and without evidence of disease progression. This duration of disease stability was notable in the context of adult-onset stage IVB Ewing sarcoma with multifocal osseous metastatic disease^4,20^.

### Clinical outcome and safety

After completion of multimodal therapy, the patient had stable disease by radiographic criteria. Peptide vaccination was initiated on 2 December 2022, 15 days after completion of radiotherapy, with a priming phase of four vaccinations within seven days followed by 19 booster vaccinations over 26 months, resulting in 23 vaccinations in total by the February 2025 manuscript data cut-off. The full vaccination schedule is provided in the Supplementary Information. Vaccination was well tolerated throughout, with only grade 1 local skin reactions including erythema, swelling, and induration. No systemic vaccine-associated adverse events or clinically relevant immune-mediated toxicities were observed. LDH remained within the institutional reference range during longitudinal monitoring up to the February 2025 data cut-off, measuring 171 U/L at baseline and 149 U/L shortly before vaccination initiation, with a maximum value of 246 U/L. TSH measurements showed no persistent laboratory abnormality suggestive of immune-mediated thyroid disease. An isolated low TSH value had been recorded before vaccination initiation during multimodal therapy. In the absence of clinical or persistent laboratory evidence of thyroid dysfunction, thyroid autoantibody testing was not performed.

**Fig. 1:**
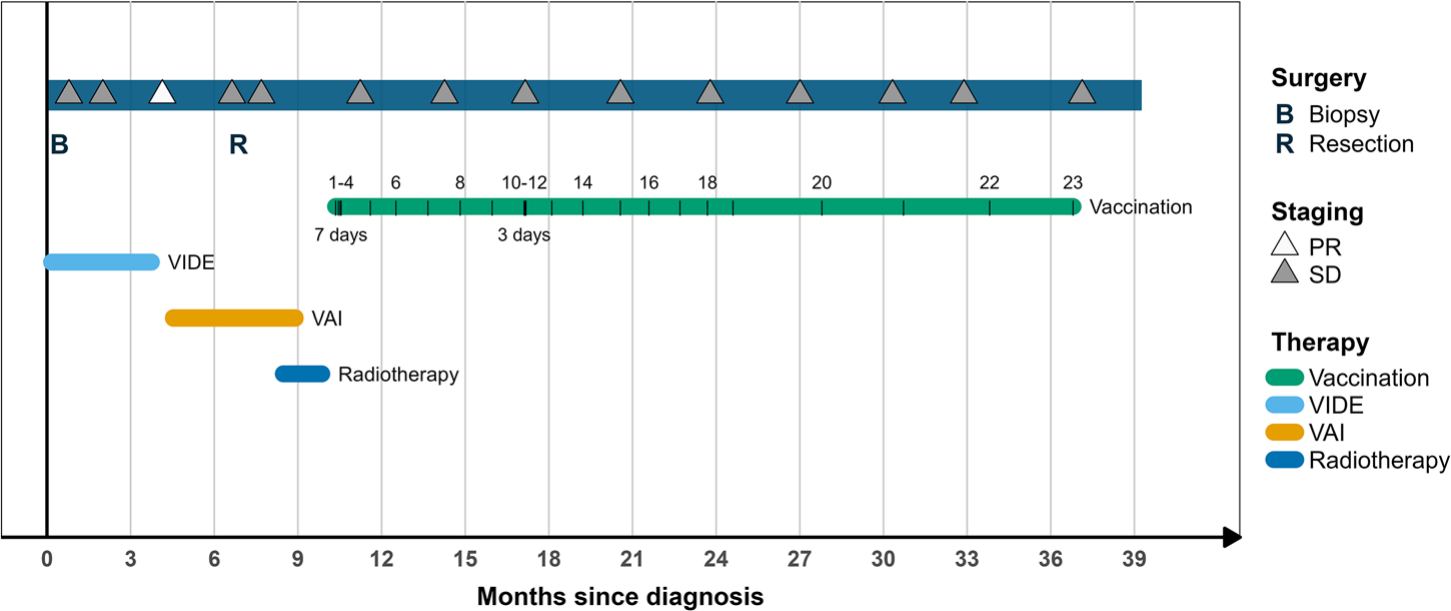
Clinical course and treatment timeline. Schematic representation of the patient’s clinical course from initial diagnosis (January 2022) to manuscript data cut-off (February 2025). The timeline depicts induction VIDE chemotherapy (six cycles), surgery of the proximal humerus with endoprosthesis implantation, adjuvant VAI chemotherapy (six cycles, three before and three after surgery), consolidative radiotherapy to the primary tumor region and osseous metastatic sites, and initiation of EWSR1–FLI1 peptide vaccination. Vaccination consisted of a priming cycle of four doses followed by 19 booster doses over 26 months. Disease status at each imaging assessment is indicated (PR, partial response; SD, stable disease). ECOG performance status remained 0 throughout follow-up. The patient experienced only grade 1 local injection-site reactions, without systemic vaccine-associated toxicities. The figure was created by the authors using the ggplot2 package, version 4.0.1, in R.

During follow-up, serial imaging with CT of the neck, chest, abdomen, and pelvis and MRI of the primary site demonstrated ongoing disease stability. Staging in January 2023 showed stable disease without new metastatic lesions. Whole-body MRI in March 2023 no longer showed clearly recognizable osseous metastases, and subsequent imaging in July, October, and January 2024 remained without evidence of local recurrence or newly detectable metastatic lesions. At the February 2025 manuscript data cut-off, more than three years after initial diagnosis and 26 months after initiation of peptide vaccination, the patient remained clinically well with ECOG performance status 0 and without evidence of recurrence or new metastatic lesions. This durable disease stability is notable in the context of adult-onset stage IVB Ewing sarcoma with multifocal osseous metastatic disease^20^. The clinical course and vaccination schedule are summarized in Fig. 1.

### Vaccine-induced T-cell responses

Peripheral blood mononuclear cells were collected prior to the first vaccination and at seven longitudinal timepoints during treatment. Antigen-specific T-cell responses were assessed following in vitro peptide stimulation and intracellular cytokine staining (ICS). Responses were defined as polyfunctional CD4⁺ or CD8⁺ T-cells co-expressing at least two functional markers (CD154, IFN-γ, TNF-α, IL-2), with a stimulation index (SI) ≥ 2 considered positive^14–16^.

At baseline, no EWSR1–FLI1–specific responses were detectable. By month 7, polyfunctional CD4⁺ T-cell responses emerged against peptides E1 (0.4% of CD4⁺ T-cells, SI 2.1) and E4 (0.7%, SI 2.2). At month 17, responses had broadened to include peptides E1, E3, and E4, with frequencies ranging from 0.7–1.6% of CD4⁺ T-cells and stimulation indices consistently above 2. By month 26, polyfunctional responses were observed against all four peptides (E1–E4), with the strongest response directed against peptide E4 (17.7% of CD4⁺ T-cells, SI 82.7). Although a single CD8⁺ response was transiently detectable, the vaccine-induced immunity was dominated by durable CD4⁺ T-cell activity.

**Fig. 2:**
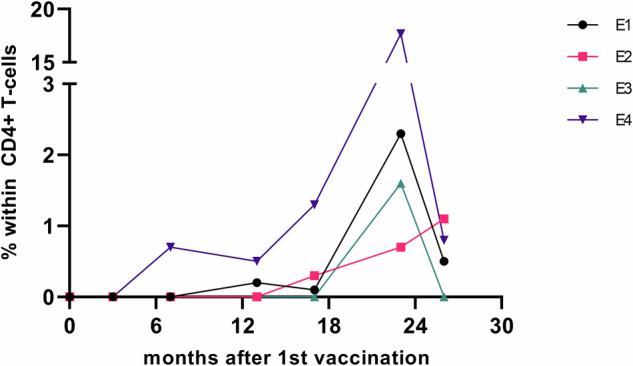
Longitudinal vaccine-induced CD4⁺ T-cell responses. Polyfunctional CD4⁺ T-cell responses to the four EWSR1–FLI1 peptides (E1–E4) were assessed in peripheral blood by in vitro peptide stimulation and intracellular cytokine staining. Responses were defined as cells co-expressing ≥2 functional markers (CD154, IFN-γ, TNF-α, IL-2) with stimulation index (SI) ≥ 2. Graphs depict frequencies of polyfunctional CD4⁺ T-cells specific for each peptide across seven timepoints. No responses were detected prior to vaccination. By month 7, responses to peptides E1 and E4 emerged. By month 17, responses broadened to E1, E3, and E4. At month 26, polyfunctional CD4⁺ T-cell responses were detectable against all four peptides, with the strongest response to peptide E4. Data represent percentages of all CD4⁺ T-cells after subtraction of mock-stimulated controls. Raw values and gating details are provided in the Supplementary Information. The figure was created by the authors using GraphPad Prism, version 8.4.3 (GraphPad Software, Boston, Massachusetts, USA).

**Fig. 3:**
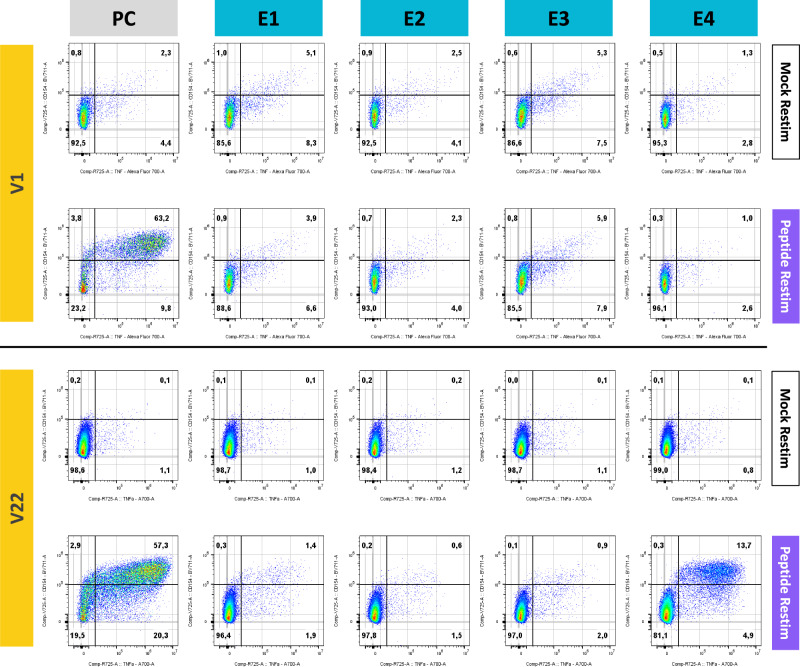
Representative flow-cytometry plots of peptide-specific T-cells. Flow-cytometry dot plots illustrating polyfunctional CD4⁺ T-cell responses prior to vaccination (V1) and after 22 vaccinations (V22). Cells were either mock-stimulated (negative control, upper row), stimulated with each of the four EWSR1–FLI1 peptides (E1–E4, lower row), or stimulated with CytoStim (Miltenyi Biotec) (positive control, PC). Shown are representative plots gated on CD4⁺ T-cells, with TNF on the x-axis and CD154 on the y-axis. Numbers indicate frequencies within all CD4⁺ T-cells. Robust vaccine-induced responses are evident after vaccination, while absent before treatment. Raw values and gating details are provided in the Supplementary Information. The figure was created by the authors using FlowJo™ Software, version 10.10.1 (BD Life Sciences).

Representative flow-cytometry plots confirmed polyfunctional cytokine co-expression and validated the specificity of responses compared to mock-stimulated controls (Fig. 3). Longitudinal analysis demonstrated persistence of vaccine-induced CD4⁺ T-cell immunity for more than two years, consistent with the generation of durable immunological memory. Longitudinal kinetics are shown in Fig. 2, representative plots in Fig. 3.

## Discussion

Metastatic Ewing sarcoma remains one of the most challenging pediatric and young adult malignancies, with five-year survival rates below 30% despite intensive multimodal therapy^2,4^. Long-term progression-free survival beyond two years is rare^20^, particularly in adult patients with multifocal osseous involvement, underscoring the urgent need for innovative therapeutic strategies.

The EWSR1–FLI1 fusion protein is a pathognomonic driver event in Ewing sarcoma. Because the fusion breakpoint generates tumor-specific neoepitopes absent from healthy tissues, it represents a rational target for precision immunotherapy^11^. Prior attempts to target this fusion used dendritic cell-based vaccines or irradiated tumor cell vaccines secreting GM-CSF, which demonstrated feasibility but were limited by logistical complexity^12,21^. The present report highlights a comparatively simple, scalable approach using pre-synthesized, off-the-shelf peptides spanning the EWSR1–FLI1 type 1 breakpoint, administered with GM-CSF and topical imiquimod as adjuvants.

Immunomonitoring revealed that vaccination induced robust, polyfunctional CD4⁺ T-cell responses against all four peptides, absent prior to treatment, emerging by month 7, and persisting for more than two years. Polyfunctional responses were defined by co-expression of at least two cytokines/activation markers (CD154, IFN-γ, TNF-α, IL-2), consistent with established definitions of vaccine-induced immunity^14–16^. The dominance of CD4⁺ responses and the relative absence of CD8⁺ reactivity may reflect the class II binding properties of the selected peptides and limited class I presentation in Ewing sarcoma.

Clinically, durable tumor control was observed for more than 26 months after vaccination initiation. This duration of disease stability is notable in the context of adult-onset stage IVB Ewing sarcoma with a large proximal humeral primary tumor, skip metastases, and multifocal osseous metastatic disease^20^. The patient had received intensive multimodal therapy before vaccination, including anthracycline- and alkylator-based VIDE induction chemotherapy, VAI consolidation chemotherapy, surgery, and consolidative radiotherapy to the primary tumor region and all known osseous metastatic sites. Therefore, the relative contribution of vaccination to long-term disease control cannot be separated from the effects of prior chemotherapy, surgery, and radiotherapy in this single case. Nevertheless, the temporal concordance of durable clinical stability with the emergence and persistence of broad polyfunctional EWSR1–FLI1-specific CD4⁺ T-cell responses supports the hypothesis that vaccine-induced immunity may have contributed to prolonged tumor control.

Several limitations must be acknowledged. This report describes a single patient, and causality between peptide vaccination and long-term disease control cannot be established. The observed clinical stability may have been influenced substantially by prior intensive multimodal therapy, including chemotherapy, surgery, and radiotherapy. Furthermore, the patient had radiographically stable disease before initiation of peptide vaccination, which limits interpretation of vaccine-specific clinical efficacy. TCR clonotyping, direct cytotoxicity assays, and functional validation of tumor recognition were not performed, which would have strengthened the mechanistic link between vaccination and the observed immune responses. The atypical late peak observed for peptide E4 may represent biologic variability or recall expansion, but requires cautious interpretation. Importantly, our assay used IL-2/IL-7 supplementation for long-term expansion of rare vaccine-induced T-cells, a protocol previously used in cancer vaccine studies^14–16^. Immune-mediated toxicity monitoring relied on clinical assessment and routine laboratory testing. Thyroid autoantibody testing was not performed because there was no clinical or persistent laboratory evidence of thyroid dysfunction suggestive of immune-mediated thyroid disease. A transient episode of diarrhea with intermittent blood admixture and nonspecific colitis occurred after completion of chemoradiotherapy and before initiation of peptide vaccination. No recurrent colitis or vaccine-associated gastrointestinal immune-mediated toxicity was observed during vaccination follow-up. Overall, vaccination was associated only with transient grade 1 local injection-site reactions and no systemic vaccine-associated toxicity. The absence of systemic immune-related toxicities and the practicality of off-the-shelf peptide synthesis further underscore the translational potential of this approach.

The present approach should be viewed within the broader landscape of peptide-based tumor vaccine development. Recent advances in peptide vaccine platforms include not only soluble synthetic peptides, but also peptide assemblies designed to improve antigen stability, antigen-presenting-cell uptake, lymph-node delivery, and co-delivery of immunostimulatory components^22^. In contrast to such engineered peptide-assembly platforms, the vaccine used in this case consisted of four overlapping soluble 17-mer peptides directly spanning the patient’s EWSR1–FLI1 type 1 fusion breakpoint, administered with local immune adjuvants. Other molecular targeting strategies, including aptamer-based theranostic platforms, are also being explored in oncology for tumor-selective recognition, imaging, and therapeutic payload delivery^23^. However, these nucleic-acid-based targeting approaches are mechanistically distinct from the fusion-derived peptide vaccination strategy described here. Together, these developments illustrate the expanding range of molecularly guided immunotherapeutic and theranostic strategies in cancer, while underscoring the specific rationale of directly targeting recurrent oncogenic fusion breakpoints in sarcoma.

In summary, this case provides first-in-human translational evidence that vaccination with fusion-derived peptides can induce durable polyfunctional immune responses and long-lasting clinical stability in metastatic Ewing sarcoma. These findings support prospective evaluation of fusion breakpoint–derived vaccines in controlled clinical trials and highlight recurrent gene fusions as actionable precision immunotherapy targets in sarcomas.

## Methods

### Vaccine design and peptide synthesis

The amino acid sequence spanning the type 1 EWSR1–FLI1 fusion breakpoint (EWSR1:NM_001163285.2 Exon 7 – FLI1:NM_002017.5 Exon 6) was used to generate four overlapping 17-mer peptides by a sliding-window approach, limiting contiguous residues from either fusion partner to a maximum of ten. To ensure broad applicability, the vaccine was designed without HLA restriction. The sequences (E1–E4) are listed in Table 1. Peptides were synthesized by solid-phase peptide synthesis and purified to >95% purity (Intavis Peptide Services, Tübingen, Germany). Lyophilized peptides (HCl salt) were dissolved in water (Aqua ad iniectabilia; BBraun, Melsungen, Germany) + 33% dimethylsulfoxide (Miltenyi, Bergisch Gladbach, Germany). Peptides were mixed and sterile-filtered through a PTFE-membrane filter (Millex-LG sterile filter; Millipore) and bottled in glass vials (Thermo Scientific). The resulting peptide cocktails were controlled for identity and purity of contained peptides as well as sterility and absence of endotoxins, followed by quality control/quality assurance release. Vaccine vials were stored at −80 °C. Details of peptide batches and release testing are provided in the Supplementary Information.

**Table 1:** EWSR1–FLI1 fusion peptides used in tumor peptide vaccination

E1	SQQSSSYGQQNPSYDSV	Fusion EWSR1:NM_001163285.2 Exon 7– FLI1:NM_002017.5 Exon 6
E2	QQSSSYGQQNPSYDSVR	Fusion EWSR1:NM_001163285.2 Exon 7– FLI1:NM_002017.5 Exon 6
E3	QSSSYGQQNPSYDSVRR	Fusion EWSR1:NM_001163285.2 Exon 7– FLI1:NM_002017.5 Exon 6
E4	SSSYGQQNPSYDSVRRG	Fusion EWSR1:NM_001163285.2 Exon 7– FLI1:NM_002017.5 Exon 6

This table lists the four peptides (E1–E4) derived from the aberrant EWSR1–FLI1 fusion protein used in the off-the-shelf tumor peptide vaccine to stimulate T-cell responses in the treatment of metastatic Ewing sarcoma.

### Vaccination protocol

Two peptide batches (14 vials each) were manufactured and used sequentially. Each vial contained 0.5 mL peptide solution (0.8 mg/mL per peptide; total dose 1.6 mg). The entire vial content was administered intracutaneously into abdominal tissue, followed by subcutaneous injection of 83 µg sargramostim and superficial application of imiquimod (50 mg/g creme) as adjuvants. The vaccination schedule included a priming cycle of four doses within seven days, followed by 19 additional booster vaccinations over 26 months (23 in total). Adverse events were recorded and graded according to CTCAE v5.0. The complete vaccination timeline, batch information, and side effect documentation are provided in the Supplementary Information.

### Immune monitoring

Detection of vaccine-induced, neoantigen-specific T-cells was performed as previously described^24^. This assay primarily detects functional, non-anergic memory T-cells in an HLA-independent manner^25^. Peripheral blood mononuclear cells (PBMCs) were collected before the first vaccination and at seven longitudinal timepoints. After thawing, PBMCs were stimulated with each peptide (5 µg/mL) in the presence of IL-2 (10 IU/mL) and IL-7 (10 ng/mL) for 12 days, then re-stimulated with the corresponding peptide or mock control. Intracellular cytokine staining (ICS) was used to detect polyfunctional T-cells, defined as cells co-expressing ≥2 markers among CD154, IFN-γ, TNF-α, and IL-2. Responses were quantified as stimulation index (SI), calculated as the ratio of polyfunctional T-cells in peptide-stimulated versus mock-stimulated conditions, with SI ≥ 2 considered positive.

The flow-cytometry panel included CD3, CD4, CD8, CD154, IFN-γ, TNF-α, IL-2, and a viability dye (reagents listed in Supplementary Information). For analysis, cells were sequentially gated on lymphocytes, singlets, live CD3⁺ T-cells, and then separated into CD4⁺ and CD8⁺ subsets. Co-expression of functional markers was assessed within these populations. Mock-stimulated and fluorescence-minus-one controls informed positivity thresholds. CytoStim (Miltenyi Biotec) served as positive control.

### Ethics approval and consent to participate

This report describes an individual therapeutic attempt performed in Germany. According to local regulatory requirements for individual therapeutic attempts, as reflected in the statement WD 9 - 3000 - 083/23 of the German Bundestag, formal institutional review board approval was not required for the individual therapeutic use of the peptide vaccine. According to the Professional Code for Physicians, publication of case reports does not require review by an ethics committee (Institutional Review Board of the Faculty of Medicine of the University of Tübingen and the University Hospital Tübingen, reference no. 280/2025BO2). The therapeutic procedure was conducted in accordance with the Declaration of Helsinki. The patient provided written informed consent for peptide vaccine treatment and for the scientific use and publication of clinical, molecular, and immunological data in anonymized form. The patient did not receive compensation.

## Supplementary information

Supplementary information

## Data Availability

The datasets generated and/or analyzed during the current study are not publicly available due to patient privacy, data protection regulations, and the potentially identifiable nature of rare-disease clinical, genomic, and immunological data, but are available from the corresponding author on reasonable request and subject to applicable institutional, ethical, and legal restrictions.
