# Supplementary material for: Durable clinical and immunologic response to an off-the-shelf EWSR1–FLI1 peptide vaccine in metastatic Ewing sarcoma
Source: NPJ Precis Oncol. 2026 Aug 8;10:305. doi: 10.1038/s41698-026-01642-4 (PMC13452805; doi:10.1038/s41698-026-01642-4)
Supplement: Supplementary file 1 [file 41698_2026_1642_MOESM1_ESM.pdf]

Supplementary Information

Supplementary Note 1. Peptide Sequence Specificity

The peptides were designed to exactly match the canonical type-1 EWSR1–FLI1 (EWSR1:NM_001163285.2 Exon 7 – FLI1:NM_002017.5 Exon 6) breakpoint sequence. The patient harbored the same fusion, enabling off-the-shelf administration without individualized peptide synthesis.

Supplementary Note 2. Timeline of radiotherapy and vaccination initiation

Consolidative radiotherapy was completed on 17 November 2022, as described in the main manuscript. The first peptide vaccination was administered on 2 December 2022, corresponding to an interval of 15 days after completion of radiotherapy.

Supplementary Table 1. Vaccination Schedule and Peptide Dosing

No	Date	Batch	Adjuvant / Immune response modifier	Adverse events
1	02.12.2022	A	Sargramostim (83 µg) and imiquimod	local skin reaction, CTCAE v5.0 grade 1
2	05.12.2022	A	Sargramostim (83 µg) and imiquimod	local skin reaction, CTCAE v5.0 grade 1
3	07.12.2022	A	Sargramostim (83 µg) and imiquimod	Local skin reaction, CTCAE v5.0 grade 1,<br>local pain
4	08.12.2022	A	Sargramostim (83 µg) and imiquimod	local skin reaction, CTCAE v5.0 grade 1
5	09.01.2023	A	Sargramostim (83 µg) and imiquimod	local skin reaction, CTCAE v5.0 grade 1, redness, swelling
6	06.02.2023	A	Sargramostim (83 µg) and imiquimod	moderate local skin reaction, redness, slight swelling
7	13.03.2023	A	Sargramostim (83 µg) and imiquimod	local skin reaction, CTCAE v5.0 grade 1
8	17.04.2023	A	Sargramostim (83 µg) and imiquimod	local skin reaction, CTCAE v5.0 grade 1, swelling, slight redness
9	22.05.2023	A	Sargramostim (83 µg) and imiquimod	moderate local skin reaction, CTCAE 1
10	26.06.2023	A	Sargramostim (83 µg) and imiquimod	moderate local skin reaction, CTCAE 1
11	27.06.2023	A	Sargramostim (83 µg) and imiquimod	local skin reaction, CTCAE v5.0 grade 1
12	28.06.2023	A	Sargramostim (83 µg) and imiquimod	local skin reaction, CTCAE v5.0 grade 1
13	26.07.2023	A	Sargramostim (83 µg) and imiquimod	local skin reaction, CTCAE v5.0 grade 1
14	29.08.2023	A	Sargramostim (83 µg) and imiquimod	local skin reaction, CTCAE v5.0 grade 1
15	09.10.2023	B	Sargramostim (83 µg) and imiquimod	local skin reaction, CTCAE v5.0 grade 1

16	09.11.2023	B	Sargramostim (83 µg) and imiquimod	local skin reaction, redness, swelling, CTCAE 1
17	13.12.2023	B	Sargramostim (83 µg) and imiquimod	local skin reaction, redness, swelling, CTCAE 1
18	12.01.2024	B	Only imiquimod	local skin reaction, redness, swelling, CTCAE 1
19	09.02.2024	B	Sargramostim (83 µg) and imiquimod	local skin reaction, redness, swelling, CTCAE 1
20	16.05.2024	B	Only imiquimod	local skin reaction, redness, swelling, CTCAE 1
21	13.08.2024	B	Only imiquimod	local skin reaction, redness, swelling, CTCAE 1
22	15.11.2024	B	Only imiquimod	typical local reaction, CTCAE 1
23	14.02.2025	B	Only imiquimod	typical local reaction, CTCAE 1

Supplementary Table 2. Peptide vaccine batch and release information

Peptide Vaccines - Batch A

Batch	Produced	Released	Sample ID	Vials produced	Comment
A	19.10.2022	02.12.2022	100799_2	14	

No.	Sequence	Variant	HLA
E1	SQQSSSYGQQNPSYDSV	0	HLA class II
E2	QQSSSYGQQNPSYDSVR	0	HLA class II
E3	QSSSYGQQNPSYDSVRR	0	HLA class II
E4	SSSYGQQNPSYDSVRRG	0	HLA class II

Peptide Vaccines - Batch B

Batch	Produced	Released	Sample ID	Vials produced	Comment
B	06.09.2023	29.09.2023	100799_2	14	

No.	Sequence	Variant	HLA
E1	SQQSSSYGQQNPSYDSV	0	HLA class II
E2	QQSSSYGQQNPSYDSVR	0	HLA class II
E3	QSSSYGQQNPSYDSVRR	0	HLA class II
E4	SSSYGQQNPSYDSVRRG	0	HLA class II

Peptide dose per vaccination:

- 0.8 mg/mL per peptide (total of 4 peptides)
- Injection volume: 0.5 mL
- *Total peptide content per dose: 1.6 mg (0.4 mg of each peptide)*

Supplementary Note 3. Rationale for early vaccination

The initial vaccinations (V1–V4) were administered 15 days after completion of consolidative radiotherapy, as described in the main manuscript; no concurrent chemotherapy was administered. Despite expected transient immune suppression, the goal was to initiate immunotherapy at the earliest feasible timepoint. The rationale included the hypothesis that radiochemotherapy-induced tumor cell death may enhance antigen availability, fostering synergistic vaccine effects. As systemic immune responses develop over several weeks, early intradermal administration allows the immune priming process to begin concurrently.

Supplementary Note 4. Criteria and plan for future vaccinations

Planned Monitoring & Criteria for Re-Vaccination:

- **Imaging:** As long as the patient remains in radiological complete or stable remission, no systemic therapy is given.
- **Immune monitoring:** Conducted every 6 months.
  - If a **robust immune response** (e.g., polyfunctional CD4+ T cells with SI ≥ 2) is detected, **booster vaccinations may be spaced longer apart**.
  - If **weak or absent responses** are noted, additional vaccinations may be re-initiated at closer intervals (e.g., monthly to bi-monthly).

Risk of Over-Vaccination:
Avoiding immune exhaustion is critical. Therefore, excessive or unnecessary vaccinations are consciously avoided, with decisions individualized based on combined assessment of disease status, prior immune response, and toxicity profile.

Supplementary Note 5. Flow cytometry and immunomonitoring methodology

Antibody Panel for ICS:

- CD3 - BV (Brilliant Violet)785 (clone UCHT1); CD4 – FITC (clone RPA-T4); CD8 – APC-Cy7 (clone SK1); CD154 – BV711 (clone 24-31); IFN-γ – BV421 (clone 4S.B3); IL-2 – PE-Cy7 (clone MQ1-17H12); TNF – Alexa700 (clone Mab11) (all BioLegend).Live/Dead discrimination: Zombie Aqua (BioLegend).
- Flow cytometer: NovoCyte 3005R (Agilent)

Supplementary Figure 1. Flow-cytometry gating strategy

We included only cells that were constantly measured over time (1; Forward-scatter (FSC)-H versus Time). Herein, single (2; FSC-A versus FSC-H), viable (3; Zombie Aqua-negative cells), lymphocytes (4; FSC-A versus Side-scatter (SSC)-A) and CD3+ T-cells (5) were selected. CD3+ T-cells were further discriminated in CD4+ or CD8+ T-cells (6). Within both CD4+ (7-10) and CD8+ T-cells (11-14), we

determined the production/expression of the functional markers IFN-γ (7, 11), TNF (8, 12), IL-2 (9, 13) and CD154 (10, 14).

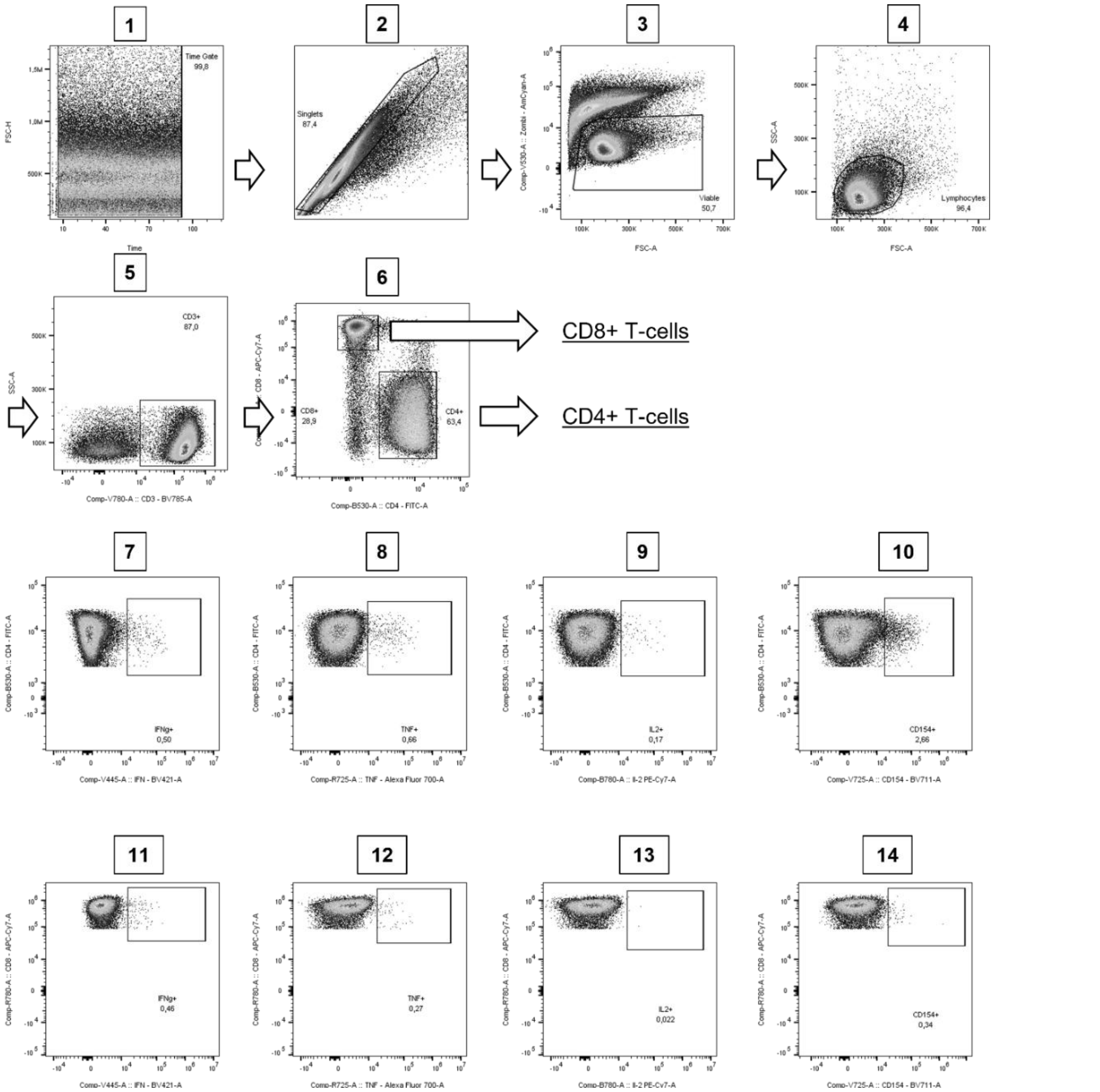

Stimulation & Controls:

- PBMCs were thawed and rested overnight.
- After washing, PBMCs were resuspended in TexMACS medium (Miltenyi Biotec) supplemented with 1% Penicillin/Streptomycin. 2.5 x 10^6 cells per well were seeded in a 48 well-plate
- Cells were stimulated with HLA class II peptides (5 µg/mL). We separately tested E1 to E4.
- After 24 hours of incubation, IL-2 (10 IU/mL) and IL-7 (10 ng/mL) were added
- Medium was changed at day 4, 6, 8, and 11.
- Expanded T cells were collected, washed and re-seeded in two wells of a 96-well plate
- One well is restimulated with the corresponding peptide 5 µg/mL; the second well was mock-restimulated with medium only.
- After addition of Golgi inhibitors (1 µL/mL; Golgi Plug, BD Biosciences), cells were additionally incubated for 14 hours
- PBMCs incubated for 12 days in medium only and then stimulated with CytoStim (10 µL/mL; Miltenyi Biotec) served as positive control

Supplementary Table 3. Longitudinal immune-monitoring summary

Date	No	Sequence	Gene	SI CD4	SI CD8	CD4 % cells positive	CD8 % cells positive
26.07.2023	E1	SQQSSSYGQQNPSYDSV	0		2.10		0.40
26.07.2023	E2	QQSSSYGQQNPSYDSVR	0				
26.07.2023	E3	QSSSYGQQNPSYDSVRR	0				
26.07.2023	E4	SSSYGQQNPSYDSVRRG	0	2.20		0.70	
09.10.2023	E1	SQQSSSYGQQNPSYDSV	0	1.06	1.00	-0.70	-0.08
09.10.2023	E2	QQSSSYGQQNPSYDSVR	0	1.20	0.82	-0.03	-0.05
09.10.2023	E3	QSSSYGQQNPSYDSVRR	0	0.90	0.93	-0.31	-0.09
09.10.2023	E4	SSSYGQQNPSYDSVRRG	0	1.80	1.43	1.65	0.03
12.01.2024	E1	SQQSSSYGQQNPSYDSV	0	3.19	0.82	0.16	0.02
12.01.2024	E2	QQSSSYGQQNPSYDSVR	0	2.00	0.91	0.08	-0.08
12.01.2024	E3	QSSSYGQQNPSYDSVRR	0	1.47	1.89	-0.01	0.05
12.01.2024	E4	SSSYGQQNPSYDSVRRG	0	2.87	0.71	0.46	-0.11
13.05.2024	E1	SQQSSSYGQQNPSYDSV	0	2.75	0.72	0.09	-0.02
13.05.2024	E2	QQSSSYGQQNPSYDSVR	0	4.88	0.82	0.28	-0.02
13.05.2024	E3	QSSSYGQQNPSYDSVRR	0	4.81	0.41	0.05	-0.07
13.05.2024	E4	SSSYGQQNPSYDSVRRG	0	30.04	1.15	1.35	0.01
15.11.2024	E1	SQQSSSYGQQNPSYDSV	0	7.65	0.70	2.32	0.01
15.11.2024	E2	QQSSSYGQQNPSYDSVR	0	2.79	1.00	0.70	0.05
15.11.2024	E3	QSSSYGQQNPSYDSVRR	0	4.97	1.33	1.61	0.01

15.11.2024	E4	SSSYGQQNPSYDSVRRG	0	82.69	0.67	17.71	0.02
14.02.2025	E1	SQQSSSYGQQNPSYDSV	0	1.94	0.71	0.51	-0.03
14.02.2025	E2	QQSSSYGQQNPSYDSVR	0	2.51	0.81	1.13	-0.02
14.02.2025	E3	QSSSYGQQNPSYDSVRR	0	1.27	0.67	0.28	-0.05
14.02.2025	E4	SSSYGQQNPSYDSVRRG	0	2.95	0.38	0.82	-0.02

Longitudinal frequencies of polyfunctional CD4⁺ T cells specific for EWSR1–FLI1 peptides (E1–E4). Values represent the percentage of CD4⁺ T cells co-expressing ≥2 functional markers (CD154, IFN-γ, TNF-α, IL-2) after background subtraction (peptide minus mock). Negative values may occur due to variability in low-frequency measurements; these have been retained in the table to ensure full transparency. For graphical display in the main figures, negative values were truncated to zero. For clarity, negative percentages were plotted as 0 in figures, while raw values are retained here to facilitate auditability and re-analysis.

Supplementary Table 4. Raw flow-cytometry values

Months after vaccination	Stimulus	Sample	Time Gate	Time Gate/Singlets	Time Gate/Singlets/Viable	Time Gate/Singlets/Viable/Lymphocytes	Time Gate/Singlets/Viable/Lymphocytes/CD3+	Time Gate/Singlets/Viable/Lymphocytes/CD3+/CD4+	Time Gate/Singlets/Viable/Lymphocytes/CD3+/CD4+/CD154+	Time Gate/Singlets/Viable/Lymphocytes/CD3+/CD4+/IFN-γ+	Time Gate/Singlets/Viable/Lymphocytes/CD3+/CD4+/IL2+	Time Gate/Singlets/Viable/Lymphocytes/CD3+/CD4+/TNF+	Time Gate/Singlets/Viable/Lymphocytes/CD3+/CD8+	Time Gate/Singlets/Viable/Lymphocytes/CD3+/CD8+/CD154+	Time Gate/Singlets/Viable/Lymphocytes/CD3+/CD8+/IFN-γ+	Time Gate/Singlets/Viable/Lymphocytes/CD3+/CD8+/IL2+	Time Gate/Singlets/Viable/Lymphocytes/CD3+/CD8+/TNF+
0	CS	NC	99.21	71.74	68.41	98.19	48.98	46.79	1.53	0.69	0.39	5.16	44.12	0.64	0.13	0.08	0.82
0	CS	Restim	99.69	77.55	77.32	97.81	67.02	16.96	51.04	36.39	28.02	64.64	35.95	0.85	15.11	0.44	16.27
0	E1	NC	99.32	66.90	63.97	97.88	56.10	46.74	3.48	0.82	1.06	10.68	44.49	1.05	0.41	0.09	1.54
0	E1	Restim	98.87	66.07	64.43	97.76	54.27	46.78	2.70	0.56	0.59	8.05	44.35	1.04	0.48	0.06	1.27
0	E2	NC	99.51	68.32	66.07	97.74	56.02	49.00	1.75	0.54	0.31	5.23	44.88	0.70	0.16	0.15	0.67
0	E2	Restim	99.39	67.85	67.98	97.62	56.43	50.51	1.71	0.50	0.41	4.83	42.74	0.42	0.11	0.05	0.57
0	E3	NC	99.61	72.33	70.76	97.15	55.63	49.35	3.45	1.04	1.10	9.92	41.35	0.74	0.55	0.05	1.46
0	E3	Restim	99.74	71.91	68.98	97.21	55.88	49.95	3.85	1.03	1.21	10.56	41.39	0.61	0.62	0.06	1.84
0	E4	NC	99.62	73.42	69.26	97.97	58.83	30.57	0.93	0.21	0.30	3.10	61.86	0.29	0.37	0.25	0.56
0	E4	Restim	99.62	72.57	68.08	97.89	58.45	31.27	0.59	0.08	0.17	2.67	61.69	0.35	0.16	0.30	0.52
3	CS	NC	99.60	60.77	63.67	98.68	46.89	30.36	2.69	1.00	0.37	4.75	62.23	0.21	0.77	0.11	1.04
3	CS	Restim	99.89	61.27	65.21	98.60	50.92	19.78	58.79	45.03	12.14	66.64	28.26	0.75	8.15	0.36	9.73
3	E1	NC	99.35	61.35	55.94	98.62	50.33	33.83	3.95	1.77	0.50	7.36	59.26	0.36	0.79	0.16	1.56
3	E1	Restim	99.86	61.63	56.29	98.67	51.54	33.79	3.35	1.38	0.43	6.24	59.02	0.26	0.65	0.10	1.23
3	E2	NC	99.83	58.66	58.23	98.46	53.44	34.69	6.31	5.61	0.68	13.42	57.36	0.16	1.02	0.08	1.82

3	E2	Restim	99.64	57.64	58.25	98.35	53.96	35.18	5.14	6.50	0.53	12.71	57.29	0.14	0.90	0.11	1.47
3	E3	NC	99.39	50.80	48.64	97.88	57.97	31.48	4.34	1.85	0.53	11.04	62.92	0.43	0.54	0.08	2.02
3	E3	Restim	99.64	51.51	48.75	98.00	59.07	31.44	5.05	1.53	0.75	11.14	62.72	0.35	0.63	0.11	2.29
3	E4	NC	99.18	61.80	55.09	98.77	50.34	32.11	2.19	0.61	0.33	3.74	58.30	0.12	0.45	0.08	0.90
3	E4	Restim	99.57	59.85	56.17	98.55	49.01	31.36	3.07	1.12	0.28	5.24	59.79	0.11	0.56	0.10	1.19
7	CS	NC	100.00	71.70	43.30	97.50	61.80	23.00	0.22	0.21	0.18	0.56	63.50	0.02	0.11	0.01	0.53
7	CS	Restim	99.90	71.60	46.50	97.80	42.80	24.30	45.00	27.00	13.40	60.00	64.10	2.84	44.90	1.81	40.40
7	E1	NC	100.00	67.90	43.20	96.70	68.40	31.30	0.25	0.63	0.07	0.33	49.50	0.07	0.12	0.01	0.41
7	E1	Restim	100.00	67.80	43.80	96.50	68.10	30.10	0.25	0.81	0.06	0.39	50.20	0.08	0.17	0.03	0.76
7	E2	NC	100.00	66.40	41.00	96.90	70.30	29.20	1.71	0.93	0.13	1.99	57.30	0.04	0.49	0.03	1.07
7	E2	Restim	100.00	66.80	43.30	96.90	71.60	30.30	1.73	0.93	0.09	2.04	56.70	0.03	0.43	0.03	1.13
7	E3	NC	100.00	69.80	41.60	97.30	61.00	22.30	0.10	0.38	0.15	0.42	64.60	0.06	0.24	0.03	1.04
7	E3	Restim	99.90	69.40	42.20	97.40	62.10	23.50	0.16	0.37	0.17	0.54	64.10	0.07	0.26	0.03	1.28
7	E4	NC	100.00	65.50	33.20	96.50	70.80	30.50	0.51	1.04	0.07	0.79	52.90	0.12	0.30	0.01	0.63
7	E4	Restim	100.00	64.90	35.80	96.60	71.90	31.10	0.84	1.67	0.22	1.43	52.90	0.10	0.38	0.01	0.69
13	CS	NC	100.00	74.00	32.10	98.70	76.40	33.30	0.00	0.00	0.10	0.20	61.00	0.00	0.10	0.00	0.20
13	CS	Restim	100.00	73.30	34.10	98.70	28.70	46.90	34.50	10.90	11.40	58.00	42.40	2.40	46.50	3.00	49.10
13	E1	NC	100.00	73.00	31.20	98.50	73.40	34.60	0.10	0.00	0.10	0.30	59.40	0.00	0.10	0.00	0.20
13	E1	Restim	99.90	73.80	31.50	98.60	74.20	34.60	0.10	0.00	0.10	0.40	59.30	0.00	0.10	0.00	0.10
13	E2	NC	99.90	74.40	35.60	98.80	74.80	34.90	0.00	0.00	0.10	0.20	59.40	0.00	0.10	0.00	0.20
13	E2	Restim	100.00	74.20	35.90	98.70	73.60	34.20	0.10	0.00	0.30	0.40	59.80	0.00	0.10	0.00	0.10
13	E3	NC	99.80	75.20	35.00	98.80	72.70	34.00	0.00	0.00	0.10	0.20	60.60	0.00	0.10	0.00	0.10
13	E3	Restim	99.90	74.80	34.10	98.70	73.60	34.40	0.10	0.00	0.00	0.40	60.40	0.00	0.00	0.00	0.10
13	E4	NC	100.00	73.40	35.60	98.70	75.20	34.10	0.00	0.00	0.10	0.30	60.50	0.00	0.10	0.00	0.10
13	E4	Restim	100.00	72.80	34.60	98.70	74.50	34.20	0.60	0.00	0.30	1.70	60.40	0.00	0.10	0.00	0.20
17	CS	NC	99.80	81.10	61.60	99.50	78.90	32.70	0.60	0.00	0.00	0.90	62.50	0.00	0.10	0.00	0.20
17	CS	Restim	99.90	81.10	65.90	99.20	47.00	34.40	65.40	22.10	30.30	72.20	59.90	4.50	39.00	5.40	48.00
17	E1	NC	100.00	82.20	64.70	99.30	76.00	34.60	0.60	0.00	0.00	0.70	59.40	0.00	0.10	0.00	0.10
17	E1	Restim	99.40	81.90	64.50	99.40	76.10	34.60	2.10	0.10	0.40	2.70	59.30	0.00	0.00	0.00	0.10
17	E2	NC	99.60	80.50	61.90	99.30	76.20	33.70	0.60	0.00	0.10	0.90	59.80	0.00	0.10	0.00	0.10
17	E2	Restim	99.80	80.50	63.90	99.40	76.00	33.60	1.00	0.10	0.30	1.60	59.80	0.00	0.10	0.00	0.20
17	E3	NC	100.00	80.90	63.30	99.20	77.10	35.30	0.40	0.00	0.00	0.90	59.00	0.00	0.00	0.00	0.10
17	E3	Restim	100.00	80.60	64.70	99.20	76.10	34.80	1.30	0.00	0.20	2.30	59.40	0.00	0.10	0.00	0.10
17	E4	NC	100.00	80.00	62.40	99.30	79.90	40.00	0.30	0.00	0.00	0.70	55.00	0.00	0.00	0.00	0.10
17	E4	Restim	100.00	79.50	63.60	99.30	80.00	39.30	14.90	0.20	5.00	17.40	55.60	0.00	0.00	0.00	0.10
23	CS	NC	100.00	75.40	22.60	98.60	70.60	28.20	0.10	0.00	0.10	0.10	65.00	0.00	0.00	0.00	0.60
23	CS	Restim	100.00	73.70	22.60	98.50	42.70	33.70	38.10	7.50	14.00	57.00	55.90	2.10	16.90	1.70	53.20
23	E1	NC	100.00	74.50	23.60	98.70	70.90	27.50	0.20	0.00	0.10	0.20	63.40	0.00	0.10	0.00	0.40
23	E1	Restim	99.90	74.00	24.50	98.80	71.80	27.50	0.30	0.10	0.10	0.40	63.70	0.00	0.00	0.00	0.40
23	E2	NC	100.00	74.70	24.40	98.70	70.30	26.90	0.20	0.00	0.10	0.10	65.60	0.10	0.00	0.00	0.50
23	E2	Restim	99.80	73.80	23.50	98.70	70.70	26.60	0.20	0.00	0.10	0.20	66.10	0.00	0.00	0.00	0.40
23	E3	NC	100.00	72.70	23.20	98.70	69.80	27.80	0.20	0.00	0.00	0.20	63.80	0.00	0.00	0.00	0.50
23	E3	Restim	99.30	72.40	22.10	98.60	70.00	27.50	0.20	0.00	0.10	0.20	63.90	0.10	0.00	0.00	0.60
23	E4	NC	100.00	75.50	24.00	98.80	73.20	28.10	0.40	0.00	0.10	0.40	64.20	0.10	0.10	0.00	0.40
23	E4	Restim	100.00	75.10	23.50	98.70	71.70	28.00	0.70	0.10	0.40	0.80	64.50	0.00	0.00	0.00	0.40
26	CS	NC	99.78	81.69	56.63	98.95	85.37	41.55	0.34	0.03	0.07	0.98	52.81	0.01	0.05	0.04	0.15
26	CS	Restim	99.96	81.32	66.40	98.24	45.51	46.57	32.42	10.95	15.53	57.44	6.84	1.38	9.14	2.11	12.86
26	E1	NC	99.77	81.19	53.12	99.00	84.55	43.03	0.39	0.03	0.11	1.08	51.75	0.02	0.04	0.06	0.16
26	E1	Restim	99.89	82.07	55.38	99.11	84.50	42.15	0.75	0.02	0.29	1.38	52.75	0.02	0.05	0.01	0.15
26	E2	NC	99.67	82.09	59.76	99.07	84.44	44.66	0.53	0.05	0.13	1.32	50.19	0.04	0.03	0.05	0.11
26	E2	Restim	99.97	81.18	60.79	98.95	83.54	44.05	1.11	0.03	0.40	2.21	50.58	0.03	0.03	0.04	0.10
26	E3	NC	99.95	82.83	58.35	99.08	81.24	37.90	0.79	0.07	0.18	1.66	56.34	0.04	0.06	0.04	0.20

26	E3	Restim	99.94	82.44	60.25	99.03	80.28	37.66	0.79	0.05	0.23	2.08	56.50	0.03	0.08	0.03	0.13
26	E4	NC	99.97	80.91	54.81	98.89	82.76	39.57	0.37	0.03	0.11	1.10	54.20	0.04	0.04	0.04	0.16
26	E4	Restim	99.93	80.49	55.49	98.87	81.86	39.98	0.71	0.03	0.33	1.86	53.83	0.02	0.04	0.04	0.12

Abbreviations: CS=CytoStim, E1–E4=fusion breakpoint-spanning peptides 1 - 4; NC=negative control (mock-restimulated sample); Restim=peptide-restimulated sample. Shown are percentages within parental population; minor rounding may occur.
